# Rainfall reduction amplifies the stimulatory effect of nitrogen addition on N_**2**_O emissions from a temperate forest soil

**DOI:** 10.1038/srep43329

**Published:** 2017-02-24

**Authors:** Shicong Geng, Zhijie Chen, Shijie Han, Fang Wang, Junhui Zhang

**Affiliations:** 1 Key Laboratory of Forest Ecology and Management, Institute of Applied Ecology, Chinese Academy of Sciences, 72 Wenhua Road, Shenyang 110016, China; 2 University of Chinese Academy of Sciences, Beijing, 100039, China

## Abstract

Soil is a significant source of atmospheric N_2_O, and soil N_2_O emissions at a global scale are greatly affected by environment changes that include continuous deposition of atmospheric nitrogen and changing precipitation distribution. However, to date, field simulations of multiple factors that control the interaction between nitrogen deposition and precipitation on forest soil N_2_O emissions are scarce. In this study, we conducted a 2-year continuous assessment of N_2_O emissions from November 2012 to October 2014 at a nitrogen addition and rainfall reduction manipulation platform in an old broad-leaved Korean pine mixed forest at Changbai Mountain in northeastern China. We found that N_2_O emissions from control plots were 1.25 ± 0.22 kg N_2_O-N ha^−1^ a^−1^. Nitrogen addition significantly increased N_2_O emissions, with the emission factor of 1.59%. A 30% reduction in rainfall decreased N_2_O emissions by 17–45%. However, in combination, nitrogen addition and rainfall reduction increased N_2_O emissions by 58–140%, with the emission factor of 3.19%, and had a larger promotional effect than the addition of nitrogen alone. Our results indicated that drought slightly decreases forest soil N_2_O emission; however, with increasing deposition of atmospheric N in temperate forest soils, the effect of drought might become altered to increase N_2_O emission.

As a component of the nitrogen cycle, N_2_O has attracted significant attention because N_2_O (1) is a long-lived greenhouse gas with an atmospheric lifetime of 114 years and a 100-year global warming potential of 298 relative to CO_2_^1^, (2) acts as a catalyst to deplete stratospheric ozone^2^, and (3) represents a form of nitrogen loss^3^. The atmospheric concentration of N_2_O has increased linearly by approximately 0.26% yr^−1^ over the past several decades and reached 319 ppbv in 2005^1^. Soils are the primary source of N_2_O emissions and contribute approximately 65% of the total N_2_O emissions to the atmosphere^4^. Soil microbial processes, including nitrification, denitrification and nitrifier denitrification, lead to the release of N_2_O from soils^5^,^6^, and these processes are all affected by soil temperature, soil moisture, substrate N availability, dissolved organic carbon (DOC) and soil pH^7^,^8^.

Changes in the global environment are anticipated and include continuous atmospheric nitrogen deposition^9^ and redistribution of precipitation^10^. These changes will likely strongly affect soil factors and therefore, alter N_2_O emissions from soil. Forest soils are an important source of atmospheric N_2_O^11^,^12^, and nitrogen addition experiments have been conducted to evaluate the effect of enhanced nitrogen deposition on N_2_O emissions in many forests^13^,^14^. Reductions in rainfall also reportedly affect forest soil N_2_O emissions^15^,^16^,^17^, but these results are not consistent. Some studies find that exclusion of throughfall, by decreasing soil moisture, reduces N_2_O emissions^17^,^18^; whereas other studies show that reductions in throughfall increase N_2_O emissions by increasing the soil content of DOC, which acts as an energy source for heterotrophic nitrifiers and denitrifiers^15^. Currently, field simulation experiments are rare that investigate the effects of the multifactor interaction between nitrogen addition and rainfall reduction on forest soil N_2_O emissions. Smolander *et al*.^19^ found that N mineralization is apparently inhibited more during drought in soil containing a supplemental nitrogen addition than in the control soil, indicating an interaction between drought and nitrogen addition. However, the combined effect of nitrogen addition and rainfall reduction on forest soil N_2_O emissions remains unknown.

Broad-leaved Korean pine (*Pinus koraiensis*) mixed forest covers an area of 42 334 ha in the Changbai Mountain region and is one of the dominant forest types in northeastern China. This area is exposed to high nitrogen deposition of at least 23 kg N ha^−1^ a^−1^ 20 and receives less precipitation in the summer and fall^21^. To examine the influence of an increase in nitrogen deposition, a decrease in precipitation and the effect of annual variability in temperature and precipitation on soil N_2_O emissions, both nitrogen addition and rainfall reduction were experimentally manipulated at this site. We hypothesized that rainfall reduction would amplify the stimulatory effect of nitrogen addition on N_2_O emissions, for less leaching losses of nitrogen^22^.

## Results

### Environmental conditions

**Figure 1:**
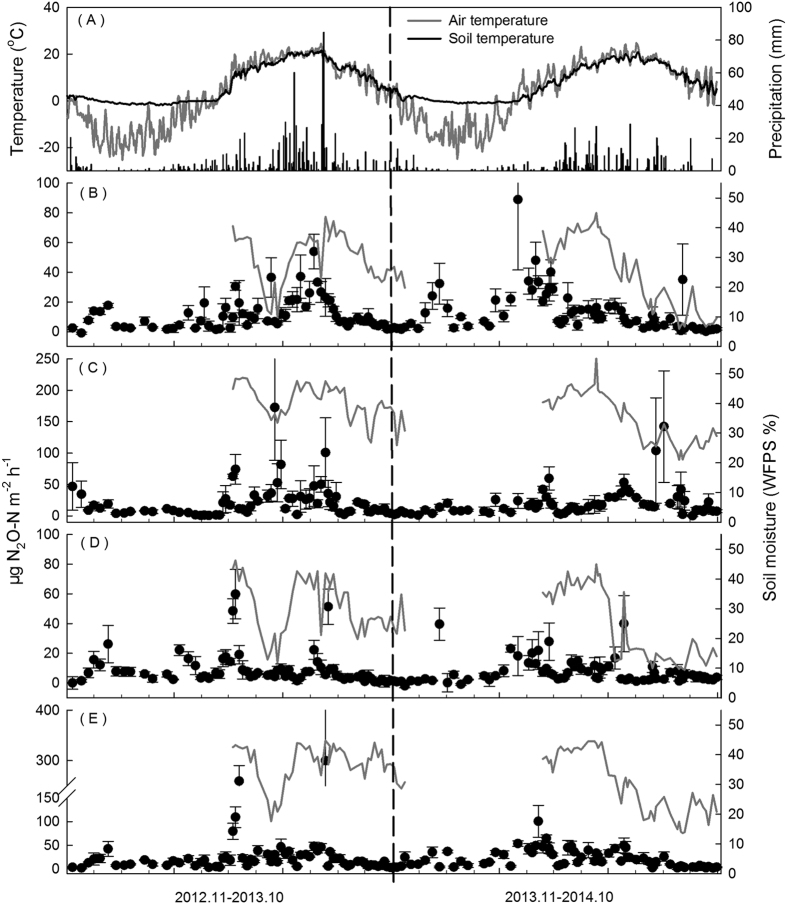
Daily mean air temperature, soil temperature (5 cm soil depth), and cumulative precipitation (**A**). N_2_O emission rates from three replicates in NF (**B**), FF (**C**), RR (**D**) and RRF (**E**) treatments measured from November 2012 to October 2014 are shown by the black circles. Soil moisture (WFPS, %) at a 5 cm soil depth was only determined during the growing seasons.

Compared with the long-term annual mean air temperature (3.2 °C) and precipitation (700 mm), the annual precipitation in 2013 was higher, and extremely heavy daily precipitation occurred. The cumulative precipitation in two days exceeded 150 mm (8/15 and 8/16; Fig. 1A). By contrast, 2014 was warmer and drier than long-term averages, with a mean air temperature of 4.3 °C and 605.6 mm of precipitation.

Significant annual and seasonal fluctuations in soil moisture were observed (Table 1). The soil moisture in all plots in 2014 was lower than that in 2013, which was consistent with the precipitation. The lowest soil moisture occurred in June 2013 and from August to October in 2014, ranging from 5.9% to 48.6% (WFPS; Fig. 1B–E). Although the magnitude of throughfall reaching the soil was reduced in the RR treatment plots, the differences observed in soil moisture between NF and RR treatments were not significant (*P* > 0.05, Table 2). In the nitrogen fertilizer treatments (FF and RRF), soil moisture was relatively higher than that in nonfertilizer treatments (NF and RR; Table 2).

Soil temperature followed a pattern similar to that of air temperature during the experiment, with significant annual and seasonal differences (*P* < 0.0001, Table 1). However, no significant differences in temperature were observed among treatments (*P* > 0.05, Table 2).

Significant differences in the seasonal dynamics of NH_4_^+^, NO_3_^−^, DOC and pH were observed (*P* < 0.0001, Table 1). However, we did not observe significant differences in soil NH_4_^+^among treatments (Table 2), whereas nitrogen addition increased concentrations of soil NO_3_^−^ and DOC compared with the control plots. Soil pH was not significantly affected by the treatments (*P* > 0.05, Table 1).

### Seasonal and annual dynamics of N_2_O emissions

For the 2 years of this study, the mean annual N_2_O emission was 1.25 ± 0.22 kg N_2_O-N ha^−1^ a^−1^ in the NF treatment; however, the emissions during 2014 (1.41 ± 0.32 kg N_2_O-N ha^−1^ a^−1^) were approximately 30% higher than those during 2013 (1.09 ± 0.13 kg N_2_O-N ha^−1^ a^−1^). The seasonal dynamics of the N_2_O emission rates in the four treatments during the two-year observation period are shown in Fig. 1B–E. The rates were generally consistent with the level of soil moisture during the growing season. In the control plots, the highest N_2_O emission rates generally occurred in July and August in 2013, when the soil moisture was high due to abundant precipitation. However, in 2014, the highest N_2_O emission rates occurred in late April and early May, when the soil experienced freeze-thaw events.

The seasonal, cumulative N_2_O emissions in the control plots were different between the two years (Table 3). In 2013, the N_2_O emissions during the growing season accounted for more than 70% of the annual emissions. However, the emissions in the three seasonal periods were approximately equal in 2014, with each period accounting for approximately 1/3 of the annual emissions.

### Effects of nitrogen addition and rainfall reduction on N_2_O emissions

**Figure 2:**
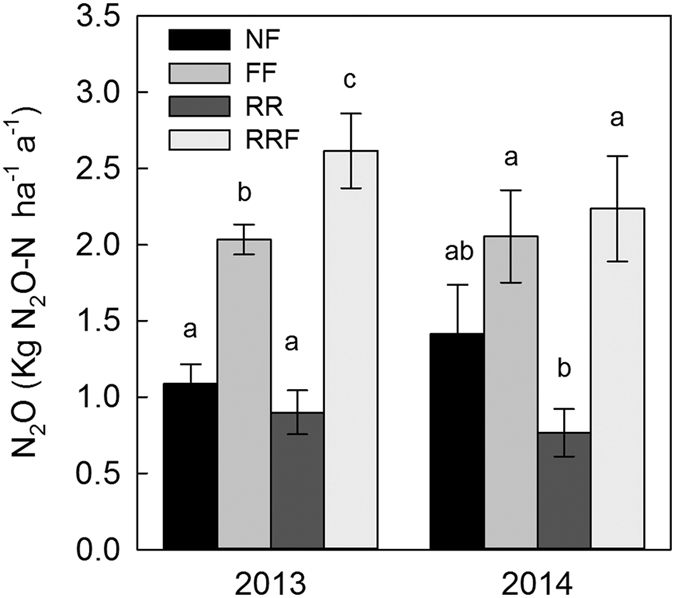
Annual cumulative N_2_O emissions in the different treatments (NF, FF, RR and RRF). Different lowercase letters indicate a significant difference at the level of 0.05 among treatments in the same year.

Nitrogen addition significantly increased the N_2_O emission rates (Table 1), and the annual emissions increased by 87% in 2013 and 45% in 2014, averaging an increase of 63% across the two years (Fig. 2). Nitrogen addition stimulated N_2_O emissions by 78% during the growing season in 2013 and by 209% in 2014. However, the effect of nitrogen addition on N_2_O emissions was not always positive. In 2014, compared with the NF treatment, the cumulative N_2_O emissions in the FF treatment decreased by 28% and 37% during the winter and freeze-thaw period, respectively (Table 3). Rainfall reduction decreased the annual N_2_O emissions by 17% in 2013 and 45% in 2014, averaging a decrease of 33% across the two years (Fig. 2), with the decrease significant (*P* < 0.05) during the growing season in 2013 and the following freeze-thaw period in 2014 (Table 3). A significant interaction was detected between nitrogen addition and rainfall reduction on N_2_O emission rates (*P* < 0.05, Table 1). Although the annual N_2_O emissions decreased in the RR treatment, the annual N_2_O emissions in RRF plots were 19% (29% in 2013 and 9% in 2014) higher than those in FF plots, with the increase significant (*P* = 0.036) in 2013 (Fig. 2). Seasonally, the cumulative N_2_O emissions in RRF plots were higher than those in the NF plots (Table 3).

The EF_N_ and EF_NP_ were 1.59% (1.89% in 2013 and 1.28% in 2014) and 3.19% (3.43% in 2013 and 2.94% in 2014), respectively, in the broad-leaved Korean pine mixed forest at Changbai Mountain, China.

### Effects of environmental factors on N_2_O emissions

Stepwise multiple linear regressions with backward elimination were used to examine the relationships between the measured soil variables and N_2_O emissions across all treatments during the entire 2014 growing season. In this model, the best predictors of observed N_2_O emissions following log-transformation were soil moisture (t = 7.68, *P* < 0.001), soil temperature (t = 4.85, *P* < 0.001) and DOC (t = −1.92, *P* = 0.059). The parameter estimations obtained from this model were determined in the following equation (residual standard error 0.67, df = 68; F-statistic 29.23, *P* < 0.001; multiple R^2^ = 0.56, Adjusted R^2^ = 0.54):


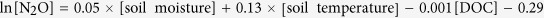


**Figure 3:**
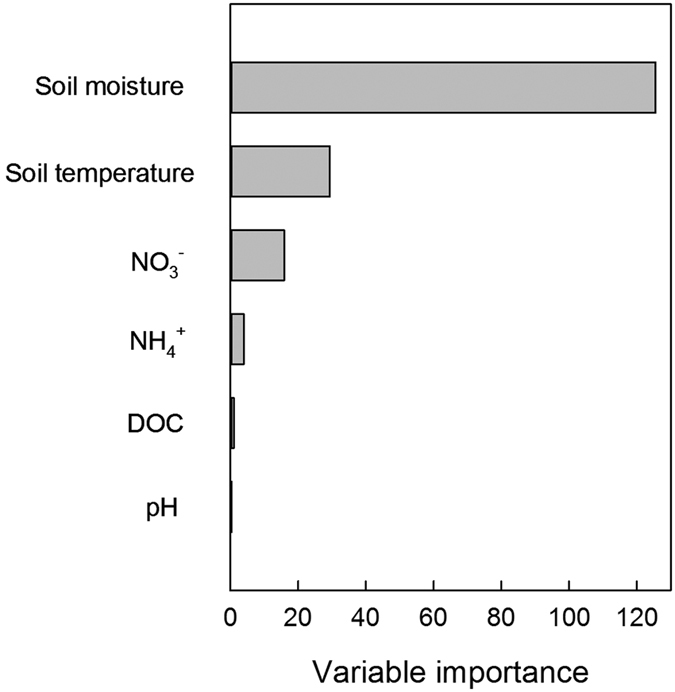
Variable importance (VIMP) for predicting N_2_O emissions.

**Figure 4:**
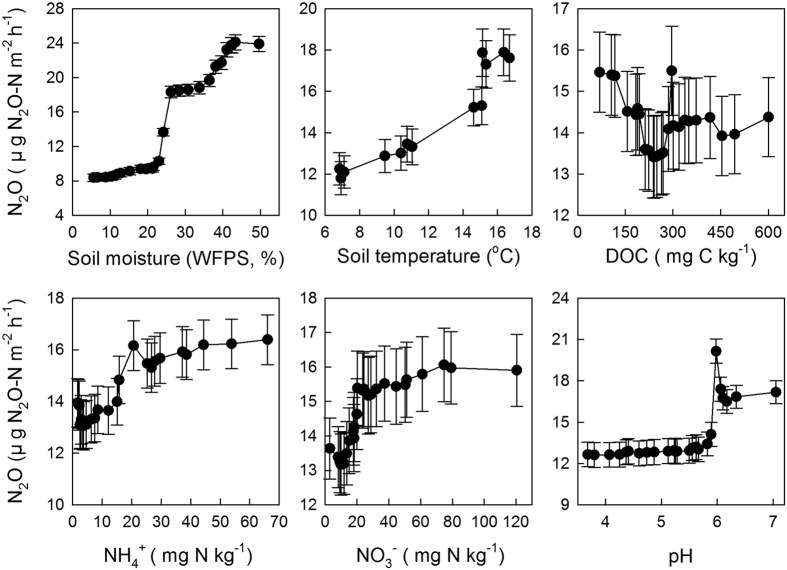
Partial correlations between N_2_O emission rates and soil variables during the growing season in 2014 in the broad-leaved Korean pine (*Pinus koraiensis*) mixed forest.

In general, soil inorganic N (NH_4_^+^and NO_3_^−^) concentration is the critical factor influencing N_2_O emissions. However, based on the stepwise multiple linear regressions, the effects of NH_4_^+^ and NO_3_^−^ on N_2_O emissions were not significant and could be eliminated. The regression model with the random ForestSRC package in the R statistical software package was also used to determine the influences of environmental factors and soil nutrients on N_2_O emissions. The values of variable importance (VIMP) in order were soil moisture > soil temperature > NO_3_^−^ > NH_4_^+^ > DOC > pH (Fig. 3). The response of N_2_O emissions for each soil variable is shown in Fig. 4. Rates of N_2_O emission increased with soil moisture and soil temperature when soil moisture ranged from 5% to 45% and soil temperature ranged from 7 °C to 17 °C. N_2_O emission rates increased rapidly when NH_4_^+^ and NO_3_^−^ concentrations were below 20 mgN kg^−1^, and then increased slowly. The N_2_O emission rates exhibited a negative relationship with DOC when the concentration was below 300 mg of C kg soil^−1^. The effect of pH on N_2_O emission rates was segmented, and the demarcation point was 6.

## Discussion

The mean annual N_2_O emission from NF plots was 1.25 ± 0.22 kg N_2_O-N ha^−1^ a^−1^, which is consistent with data reported in previous studies on N_2_O emissions for temperate forest soils^12^,^23^,^24^,^25^. However, low emissions of less than 0.1 kg N_2_O-N ha^−1^ a^−1^ 26,27 and high emissions that exceed 4 kg N_2_O-N ha^−1^ a^−1^ 28,29 have both been reported. The marked differences in emissions might be due to spatial variation, and the annual N_2_O emissions could make a large difference in the same area^30^. These large temporal and spatial variations in emissions can be attributed to two causes. First, soil N_2_O emissions are influenced by numerous factors, including precipitation, temperature, forest type, and soil texture and characteristics^7^, and small changes in these variables may result in large differences in N_2_O emissions^3^. Second, N_2_O emissions are characterized by short pulse emissions related to nitrogen deposition^31^, precipitation^32^,^33^, freeze-thaw cycles^34^ and drying-wetting events^35^. And these pulse events of N_2_O emission may be extremely important contributions to the total annual budget^30^. Field studies on N_2_O emissions often use discontinuous measurements performed with chambers over durations of weeks to months^36^,^37^, and even automated measuring systems can have observation intervals. Thus, missing pulse emission events or measuring during pulse emission events could result in underestimating or overestimating annual N_2_O emission, respectively^38^.

During the growing season, N_2_O emissions were higher in 2013 than in 2014 (*P* = 0.053), which was likely because of higher soil moisture due to abundant rain in summer 2013. However, during the non-growing seasons, particularly the freeze-thaw period, the annual N_2_O emission in 2014 was larger than that in 2013. With less snow cover and lower soil temperatures, the creation of an adverse soil environment can result in increased mortality of plant roots and soil biota^39^, which leads to the accumulation of labile soil organic carbon and nitrogen. Therefore, when this sufficient supply of soil nutrients was coupled with suitable soil moisture, a burst in N_2_O emissions occurred during the freeze-thaw period in 2014. Additionally, the result of the high N_2_O emission rates during the freeze-thaw period was likely greatly reduced soil concentrations of nitrate or carbon^33^, which then led to reduced N_2_O emissions during the growing season in 2014.

The contribution to N_2_O emissions from the nitrogen addition was significant in the broad-leaved Korean pine mixed forest, and the emission factor was 1.59%. Increases in forest soil N_2_O emissions following nitrogen addition are widely reported^13^,^14^, but the N_2_O emission factors differ by region and forest type. In this study, the N_2_O emission factor was higher than the 0.1% for a spruce forest in Germany^40^, the 0.7–0.8% for a subtropical forest in southern China^13^, and the default factor of 1% used by the IPCC^1^. In natural ecosystems, particularly those N-limited and without a history of N addition, emission levels of N_2_O are generally low and the responses to added N are weak^41^. By contrast, our study area was exposed to high levels of nitrogen deposition estimated at more than 23 kg N ha^−1^ a^−1^ 20; and the FF treatment plots received supplemental nitrogen for four years before beginning the N_2_O observations. Therefore, additional N_2_O would be emitted in this area compared with natural forest following nitrogen addition, resulting in a higher N_2_O emission factor. Compared with the NF plots, the cumulative N_2_O emission of the FF plots was significantly higher during the growing season in both years (Table 3). Pulse N_2_O emissions 2- to 4-fold greater were observed after nitrogen addition (Fig. 1C), which was consistent with previous studies conducted in the identical area^31^; the increase in emission rates persisted for approximately two weeks. These pulse emissions were the primary reason for the differences between FF and NF plots during the growing season. Additionally, soil moisture and NO_3_^−^ concentrations were higher in FF plots than those in NF plots (Table 2), which provided a more favourable environment for denitrification, leading to the increase in N_2_O emissions. In winter and the freeze-thaw period, the differences in cumulative N_2_O emissions between NF and FF plots decreased compared with that in growing season (Table 3). There are two possible explanations for this decrease in differences. First, N was only applied from May to October, and therefore, the effect of N addition likely decreased during the non-growing season. Second, the limiting factors might be soil temperature and/or soil moisture rather than soil N content during winter and the freeze-thaw period^42^.

As expected, rainfall reduction lowered the annual N_2_O emissions by 17–45%, similar to reports in previous studies^16^,^17^,^43^. However, no significant difference in soil moisture was observed between RR and NF plots, which was similar to the results for a tropical forest in which total rainfall was reduced by 25% and 50%^15^. This lack of response in soil moisture to rainfall reduction might be because precipitation is mainly concentrated in summer period, and subsurface lateral water flow reduced the effect of rainfall reduction. Although rainfall reduction had no significant effect on soil moisture; it did decrease the precipitation pulses which could cause short peak emissions of N_2_O emission^32^,^33^.

The combination treatment of nitrogen addition and rainfall reduction increased N_2_O emissions by 58–140% and had a larger stimulatory effect on emissions than the addition of nitrogen alone. A reduction in rainfall could prevent the added nitrogen from leaching out of the soil^22^. Thus, more nitrogen would remain in the soil, resulting in increased N_2_O emissions under RRF conditions compared with those under FF, as evidenced by the significantly higher soil NO_3_^−^ concentration on RRF compared to FF plots (Table 2). Because the level of precipitation in 2014 was far less than that in 2013, the protective effect of rain reduction on nitrogen would be expected to weaken, which helped to explain the difference in N_2_O emissions between RRF and FF plots between 2014 and 2013 (Fig. 2). Therefore, we concluded that a reduction in rainfall might increase the stimulatory effect of N addition on soil N_2_O emissions.

Stepwise multiple linear regressions with backward elimination indicated that soil moisture, soil temperature and DOC were the best predictors of the observed N_2_O emission patterns during the growing season in 2014. Soil moisture and soil temperature are widely reported as the best predictors of N_2_O emissions in other temperate forests^12^,^23^,^44^. Previous studies have demonstrated that a WFPS value of 60% is an important threshold because nitrification activity is the highest and denitrification activity begins to increase when the WFPS is approximately 60%^45^,^46^. In our study, soil WFPS values were slightly less than 60%, indicating that nitrification might be the most important process regulating N_2_O emissions in the broad-leaved Korean pine mixed forest. Significant positive correlations between soil temperature and N_2_O emissions were observed in this study that were consistent with those reported in other studies^23^,^44^. In our study, N_2_O emissions and DOC concentration were slightly negatively correlated. In general, as a labile nutrient, a higher concentration of DOC promotes the activities of soil biota (i.e., nitrifiers and denitrifiers), resulting in increases in N_2_O emissions. However, with the addition of external N, soil N_2_O emissions and DOC concentration might become negatively correlated^47^. On one hand, N addition could stimulate soil N_2_O emissions and consume more DOC; on the other hand, N addition could inhibit soil C mineralization and decrease soil DOC concentration^48^,^49^, as evidenced by the higher N_2_O emissions and lower DOC concentrations in FF than in NF plots.

In addition, soil inorganic N (NH_4_^+^ and NO_3_^−^) content was the essential factor influencing N_2_O emission. However, based on stepwise multiple linear regressions, the effects of NH_4_^+^ and NO_3_^−^ were not significant and could be eliminated from the model. To further investigate the influence of soil variables on N_2_O emissions, we used the regression model provided with the randomForestSRC package in the R statistical software package. Similar to the results of the stepwise multiple linear regressions, soil moisture and soil temperature were also the two most important factors influencing N_2_O emissions. However, NO_3_^−^ and NH_4_^+^ were more important factors than DOC in this model. From the partial correlation plots (Fig. 4), we observed that the N_2_O emission rates were only influenced by NO_3_^−^ or NH_4_^+^ at concentrations less than 20 mgN kg^−1^. This small range of influence might explain why inorganic nitrogen factors were eliminated by the stepwise multiple linear regressions.

## Conclusions

The response of soil N_2_O emissions to nitrogen addition and rainfall reduction was studied for two continuous years in an old broad-leaved Korean pine mixed forest at Changbai Mountain in northeastern China. We found that nitrogen addition significantly increased N_2_O emissions and rainfall reduction slightly decreased N_2_O emissions. However, the combination of nitrogen addition and rainfall reduction stimulated N_2_O emissions more than the nitrogen addition alone. This result could be explained by the reduction in rainfall preventing the added nitrogen from leaching out of the soil, and therefore, with more nitrogen remaining in the soil, N_2_O emissions increased. Our findings indicated that drought would slightly decrease forest soil N_2_O emission; however, with increasing atmospheric N deposition, drought might alter to increase the N_2_O emission in temperate forests.

## Methods

### Study site

The experiment was conducted in an old broad-leaved Korean pine (*Pinus koraiensis*) mixed forest (~200 years old) on Changbai Mountain (42°24′ N, 128°05′ E; 766 m a.s.l.) in northeastern China. The detailed description of this site can be found in Wang *et al*.^50^. The site is characterized as having a temperate, continental climate. The mean annual temperature is 3.2 °C, and the mean annual precipitation is 700 mm, primarily occurring from June to August. The soil in this region was developed from volcanic ash and is classified as a Eutric cambisol (FAO classification) containing a high content of organic matter in the organic horizon (O-horizon). The bulk density was 0.35 g cm^−3^ in the surface soil (0–10 cm), and the contents of total carbon, nitrogen and phosphorus were 11.8%, 0.9% and 0.1%, respectively.

### Experimental design

The experiment was initiated in September 2009 with the following four treatments: rainfall-reduction (RR), N-fertilization (FF), rainfall-reduction and N-fertilization (RRF) and control plots (NF). The study design included three replicates of each treatment. Thus, twelve 50 × 50 m plots with a buffer zone of at least 20 m between any two plots were established randomly within the study area. For the RR treatment, V-shaped polycarbonate (PC) of high transparency (approximately 95%) was used to prevent approximately 30% of natural throughfall. The PC was fixed on an aluminium frame ~1 m above the soil surface. For the FF treatment, ammonium nitrate (NH_4_NO_3_) solution was applied monthly (six times from May to October each year) with a sprayer. The application rate of 50 kg N ha^−1^a^−1^ was approximately double the annual total N deposition (~23 kg N ha^−1^a^−1^) in this area^20^. In the RRF treatment, throughfall was reduced by 30% and 50 kg N ha^−1^a^−1^ as NH_4_NO_3_ fertilizer was applied. The control plots received natural rainfall and atmospheric N deposition.

### N_2_O emissions

N_2_O emissions were measured using the static opaque chamber method^37^. Trapezoid-shaped chambers with the body constructed of polyvinyl chloride plastic (top: 55 × 40 cm; bottom: 47 × 32 cm; height: 30 cm) were permanently inserted into the forest soil to a depth of 5 cm, which resulted in a volume of 0.055 m^3^. Two parallel chambers were installed in each plot five months before gas sampling.

Emissions of N_2_O were measured from November 2012 to October 2014. Measurements were conducted between 8:00 and 11:00 a.m. every three to four days (8 times per month) during the growing season (April to October) and every seven to eight days (4 times per month) during the non-growing season. On each sampling, plastic tops were fitted to the chambers to ensure a gas-tight environment, and a 60-mL plastic syringe with a three-way valve was used to obtain air samples. During a 1 h incubation at 15 min intervals, five gas samples were collected from each chamber. Air temperature inside the chambers was recorded using a temperature sensor probe (JM624; Jinming Instrument CO., LTD, Tianjin, China).

All air samples were analysed within 24 h of sampling using a gas chromatograph (Agilent-7890A; Agilent Technologies, USA) equipped with an electron capture detector. The initial rates of change of the gas concentrations within the chambers were determined using significant nonlinear (exponential) or linear fitting of the five concentration observations versus enclosure time. An observation was rejected as null datum when the correlation between the gas concentrations and the enclosure time was not significant (*P* < 0.05)^51^.

### Meteorological data and soil physicochemical analyses

Meteorological data (i.e., air temperature, soil temperature and precipitation) were obtained from the Changbai Forest Ecosystem Research Station. During the non-frozen period (April through October), the soil temperature (5 cm) and soil moisture (WFPS, 0–6 cm) of each field plot were manually measured simultaneously during gas sampling using a temperature sensor probe (JM624; Jinming Instrument CO., LTD, Tianjin, China) and time-domain reflectometry (Hydro Sense II; USA), respectively. To determine the factors that influenced N_2_O emissions, soil samples were collected monthly from a depth of 10 cm in each plot using a 3 cm diameter soil auger from May to October in 2014. The soil samples were analysed for ammonium (NH_4_^+^), nitrate (NO_3_^−^), and dissolved organic carbon (DOC) concentrations and pH. Inorganic nitrogen (NH_4_^+^ and NO_3_^−^) was extracted from twenty grams of soil for 1 h in 100 mL of a 2 M potassium chloride (KCl) solution. The concentrations of NH_4_^+^ and NO_3_^−^ were determined with an automatic nitrogen analyser (AA3; BRAN & Lubbe, Nordstedt, Germany). The DOC was extracted by shaking 20 g of soil sample for 1 h in 100 mL of deionized water. The extracts were centrifuged at 6,000 rpm for 15 min. The supernatant was filtered through a 0.45 μm polyethersulfone membrane filter (Membran, Germany) before analysis with a Multi N/C 3000 analyser (Analytic Jena AG, Germany). Air-dried soils were sieved through a 2 mm mesh, and the pH was determined by electrode at a soil: water ratio of 1:1.

### Statistical analyses

All statistical analyses were conducted using the R statistical software package (version 3.2.0, Core Team 2009). Linear mixed-effects models with plot as the random factor were used to test the effects of treatment and time (i.e., month and year) on N_2_O emission rates and soil variables. Annual means for all variables were calculated for each plot and were analysed using linear mixed-effects models, with treatment and year as fixed factors and plot as the random factor. Seasonal cumulative N_2_O emissions were calculated for each plot and were analysed using linear mixed-effects models, with treatment, season and year as fixed factors and plot as the random factor. Similarly, annual cumulative N_2_O emissions were calculated for each plot and were analysed using linear mixed-effects models, with treatment and year as fixed factors and plot as the random factor.

The Shapiro test revealed that N_2_O emissions were log-distributed. After log transformation, stepwise multiple linear regressions with backward elimination using a linear model were conducted to examine the relationships between monthly mean N_2_O emissions and soil variables. We also used the randomForestSRC package (version 1.6.1) to assess the influences of soil variables on N_2_O emissions.

The N_2_O emission factors under nitrogen addition (EF_N_) and nitrogen addition with rainfall reduction (EF_NP_) for broad-leaved Korean pine mixed forest soil were calculated as follow:


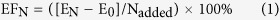



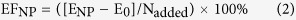


where E_N_, E_NP_ and E_0_ are the N_2_O emissions from the FF, RRF and NF plots during the same period, respectively, and N_added_ is the amount of nitrogen fertilizer applied.

## Additional Information

**How to cite this article:** Geng, S. *et al*. Rainfall reduction amplifies the stimulatory effect of nitrogen addition on N_2_O emissions from a temperate forest soil. *Sci. Rep.* **7**, 43329; doi: 10.1038/srep43329 (2017).

**Publisher's note:** Springer Nature remains neutral with regard to jurisdictional claims in published maps and institutional affiliations.

## Figures and Tables

**Table 1:** ANOVA results of the linear mixed-effects model testing the treatment, seasonal and yearly effects of experimental manipulations on soil variables.

	All treatments			Nitrogen addition			Rainfall reduction			Nitrogen addition and rainfall reduction		
	df	F	P	df	F	P	df	F	P	df	F	P
**N**_**2**_**O**												
Treatment	3	16.34	****	1	45.18	****	1	0.03		1	3.82	*
Month	11	16.96	****	11	5.63	****	11	3.92	****	11	3.20	***
Year	1	3.79	·	1	3.36	·	1	2.69		1	0.04	
**Soil moisture**												
Treatment	3	4.28	**	1	11.48	***	1	0.49		1	0.90	
Month	7	80.44	****	7	3.08	**	7	2.19	*	7	0.43	
Year	1	267.29	****	1	0.19		1	0.45		1	0.01	
**Soil temperature**												
Treatment	3	0.00		1	0.00		1	0.00		1	0.00	
Month	7	1716.93	****	7	0.09		7	0.08		7	0.09	
Year	1	18.17	****	1	1.37		1	1.41		1	1.92	
**NH**_**4**_^**+**^												
Treatment	3	0.19		1	0.12		1	0.13		1	0.97	
Month	4	16.13	****	5	2.71	*	5	1.55		5	0.66	
**NO**_**3**_^**−**^												
Treatment	3	4.10	*	1	10.00	*	1	7.94	*	1	0.36	
Month	4	14.95	****	5	2.07	·	5	1.41		5	0.34	
**DOC**												
Treatment	3	5.03	*	1	13.70	**	1	1.30		1	0.07	
Month	4	22.56	****	5	0.73		5	1.96		5	0.73	
**pH**												
Treatment	3	0.15		1	0.11		1	0.15		1	0.18	
Month	5	7.29	***	5	0.78		5	0.30		5	0.59	

^·^P < 0.1. *P < 0.05. **P < 0.01. ***P < 0.001. ****P < 0.0001.

**Table 2:** Annual means (±SE) for soil variables in different treatment plots in a broad-leaved Korean pine (*Pinus koraiensis*) mixed forest.

		NF	FF	RR	RRF
Soil moisture^†^(WFPS, %)	2013	30.17 ± 2.47 aA	41.16 ± 1.37 bA	31.06 ± 4.65 aA	37.25 ± 1.79 abA
	2014	24.15 ± 1.25 aA	34.63 ± 4.44 bA	24.40 ± 3.16 aA	30.21 ± 0.69 abB
Soil temperature^†^°C	2013	11.69 ± 0.20 aA	11.90 ± 0.00 aA	11.73 ± 0.22 aA	11.68 ± 0.20 aA
	2014	11.28 ± 0.10 aA	11.06 ± 0.01 aB	11.28 ± 0.10 aA	11.28 ± 0.10 aA
NH_4_^+^ (mg N kg^−1^)^‡^		19.99 ± 1.87 a	17.25 ± 1.78 a	17.24 ± 1.99 a	18.53 ± 1.01 a
NO_3_^−^ (mg N kg^−1^)^‡^		22.86 ± 2.71 a	31.51 ± 4.04 a	30.35 ± 1.58 a	43.08 ± 4.40 b
DOC (mg C kg^−1^)^‡^		300.08 ± 13.53 ab	236.69 ± 11.71 a	347.66 ± 36.96 b	259.32 ± 28.73 a
pH^‡^		5.27 ± 0.51 a	5.52 ± 0.26 a	5.54 ± 0.24 a	5.51 ± 0.24 a

^†^Measured across the 2013 and 2014 growing seasons. ^‡^Measured only for the growing season in 2014. Different capital letters indicate a significant difference at the level of 0.05 between years for the same treatment; different lowercase letters indicate a significant difference at the level of 0.05 among treatments in the same year.

**Table 3:** Seasonal N_2_O-N emissions (Mean ± SE, kg N_2_O-N ha^−1^) in different treatments, with the percentage contribution to annual total emissions in brackets (Mean ± SE, n = 3).

		NF	FF	RR	RRF
2013	WinterFreeze-thaw periodGrowing season	0.24 ± 0.02 (22.22) a	0.44 ± 0.11 (21.47) a	0.36 ± 0.08 (40.30) a	0.55 ± 0.11 (21.01) a
		0.12 ± 0.02 (10.83) a	0.23 ± 0.05 (11.33) b	0.21 ± 0.03 (23.27) ab	0.49 ± 0.01 (18.76) c
		0.77 ± 0.09 (70.45) a	1.37 ± 0.04 (67.19) c	0.33 ± 0.05 (36.43) b	1.57 ± 0.17 (60.23) c
2014	WinterFreeze-thaw periodGrowing season	0.49 ± 0.14 (34.42) ab	0.35 ± 0.08 (16.97) ab	0.24 ± 0.04 (31.38) a	0.63 ± 0.11 (28.11) b
		0.47 ± 0.09 (33.28) bc	0.30 ± 0.06 (14.36) ab	0.17 ± 0.06 (22.84) a	0.60 ± 0.06 (26.94) c
		0.46 ± 0.10 (32.30) a	1.41 ± 0.44 (68.67) b	0.35 ± 0.06 (45.78) a	1.00 ± 0.19 (44.94) ab

Different letters indicate a significant difference at the level of 0.05 among different treatments during the same season.
